# Generation and Characterisation of Mice Deficient in the Multi-GTPase Domain Containing Protein, GIMAP8

**DOI:** 10.1371/journal.pone.0110294

**Published:** 2014-10-17

**Authors:** Louise M. C. Webb, John C. Pascall, Lucy Hepburn, Christine Carter, Martin Turner, Geoffrey W. Butcher

**Affiliations:** Laboratory of Lymphocyte Signalling and Development, The Babraham Institute, Cambridge, United Kingdom; Duke University Medical Center, United States of America

## Abstract

**Background:**

GTPases of the immunity-associated protein family (GIMAPs) are predominantly expressed in mature lymphocytes. Studies of rodents deficient in GIMAP1, GIMAP4, or GIMAP5 have demonstrated that these GTPases regulate lymphocyte survival. In contrast to the other family members, GIMAP8 contains three potential GTP-binding domains (G-domains), a highly unusual feature suggesting a novel function for this protein. To examine a role for GIMAP8 in lymphocyte biology we examined GIMAP8 expression during lymphocyte development. We also generated a mouse deficient in GIMAP8 and examined lymphocyte development and function.

**Principal Findings:**

We show that GIMAP8 is expressed in the very early and late stages of T cell development in the thymus, at late stages during B cell development, and peripheral T and B cells. We find no defects in T or B lymphocyte development in the absence of GIMAP8. A marginal decrease in the number of recirculating bone marrow B cells suggests that GIMAP8 is important for the survival of mature B cells within the bone marrow niche. We also show that deletion of GIMAP8 results in a delay in apoptotic death of mature T cell *in vitro* in response to dexamethasone or γ-irradiation. However, despite these findings we find that GIMAP8-deficient mice mount normal primary and secondary responses to a T cell dependent antigen.

**Conclusions:**

Despite its unique structure, GIMAP8 is not required for lymphocyte development but appears to have a minor role in maintaining recirculating B cells in the bone marrow niche and a role in regulating apoptosis of mature T cells.

## Introduction

GIMAP8 is a member of the family of guanosine triphosphatases (*G*TPases) of the *im*munity-*a*ssociated *p*roteins (GIMAPs [1]). GIMAP family members are guanine nucleotide binding proteins characterised by the presence of an AIG1 domain, a domain first described in the AIG1 protein of *Arabidopsis*: the gene encoding this protein was originally identified as being involved in a defence response to bacterial invasion in plants of this species [2]. GIMAP-encoding genes are found in all vertebrates, but the presence of GIMAP/AIG1 genes in living species is generally sporadic. Thus, genes of this type have been reported in some molluscs and protists but, with the exception of *A. thaliana,* they are absent from convenient model organisms, viz, *Saccharomyces cerevisiae*, *Caenorhabditis elegans* and *Drosophila melanogaster* [1] [3] [4] [5].

In mammals there are between 7 and 8 *GIMAP* genes, found in a tight cluster [6]. Genetic association studies have implicated *GIMAP* genes in autoimmune diseases including, systemic lupus erythematosus, Behçet's disease, and type 1 diabetes [7] [8] [9] [10]–[14]. Mammalian GIMAPs are most strongly expressed in lymphoid tissue, with weaker expression seen in heart, lung, and kidney [6], [13]–[18]. *In vivo* and *in vitro* studies have indicated a role for mammalian GIMAPs in lymphoid survival and homeostasis [1]. To date, studies in rodents deficient in GIMAP1 and GIMAP5 have shown a requirement for these proteins in the survival of mature, peripheral lymphocytes [11] [12] [13], [14], [19]–[23]. In contrast, GIMAP4 is thought to have a pro-death function, since T cells from mice and rats deficient in GIMAP4 have a survival advantage when subjected to apoptotic stimuli *in vitro* [24], [25].

The mode of action for different GIMAPs remains unresolved. This is due in part to the small cytoplasmic volume within lymphocytes in which to study GIMAP location and function. Consequently, many studies have used non-lymphoid systems to look at the subcellular location and function of GIMAPs. Using immunocytochemical analysis of endogenously expressed GIMAPs in lymphoid cells, our group has shown that GIMAP1 is found in the Golgi apparatus while GIMAP5 is found in lysosomes and multivesicular bodies [26]. Work by other groups has placed GIMAP1 at the ER or on mitochondrial membranes; GIMAP5 has also been claimed to be present on mitochondria where it is suggested to associate with Bcl-xl, Bcl-2, Mcl-1 and HSC70 [27]–[31]. It is thought that GIMAP5 strengthens the association of Bcl-2 family members with HSC70 on mitochondria [27]. A role for GIMAP5 in regulating calcium signalling in T cells has also been proposed, whereby GIMAP5 promotes mitochondrial calcium accumulation [32]. The resolution of these diverse observations is awaited.

Recently, our group showed an association of GIMAP6 with the autophagy protein GABARAPL2 [33]. This suggested that GIMAP6 plays a role in autophagy, an idea supported by its relocalisation from the cytosol to LC3^+^ autophagosomes when cells were starved or treated with mTOR inhibitors. GIMAP3 has been shown to be a key regulator of mitochondrial DNA segregation within leukocytes and it has been speculated that it might function in the retromer vesicle pathway [34]. Interestingly, it has recently been shown that overexpressed GIMAP7 partially colocalised with GIMAP2 on lipid droplets and that GIMAP7 can stimulate the GTPase activity of GIMAP2 *in vitro* by heterodimerization [5], [35], [36]. It has hence been speculated that GIMAPs function in heterotypic dimers, possibly explaining why only GIMAPs 1, 2, 3, and 5 have transmembrane domains (see below [37]).

GIMAPs have been placed within the TRAFAC class of small GTPases, close to the septins [38]. They also share features with the dynamins, another TRAFAC subclass. They are composed of an N-terminal GTPase/AIG1 domain, followed by C-terminal extensions of 60–130 amino acids. GIMAP1 and GIMAP5 each contain a single C-terminal transmembrane helix, which anchors them to intracellular membranes [36]. GIMAP3, which shares 84% amino acid identity to GIMAP5, also has a C-terminal transmembrane domain but its subcellular location remains unresolved [1]. Human GIMAP2 (this GIMAP is absent from rodents) has two C-terminal hydrophobic stretches, possibly in a hairpin formation, which apparently target it to lipid droplets [36]. Remarkably, GIMAP8 has three GTPase domains, a highly unusual feature which suggests its properties/functions may differ from those of other GIMAP family members [39]. To date, little is known about the expression patterns and/or function of GIMAP8. We have addressed these questions using novel antibodies against GIMAP8 and by generating a GIMAP8 knockout mouse. Our data show that GIMAP8 is most strongly expressed in mature peripheral T and B lymphocytes. We find that deletion of GIMAP8 results in a reduction in the number of recirculating B cells found in the bone marrow. Surprisingly, we find no obvious effect on either T or B cell development or function in the absence of GIMAP8. We do, however, observe a perturbed apoptosis of GIMAP8-deficient T cells *in vitro*.

## Results

### Expression of GIMAP8 in developing T and B lymphocytes

**Figure 1:**
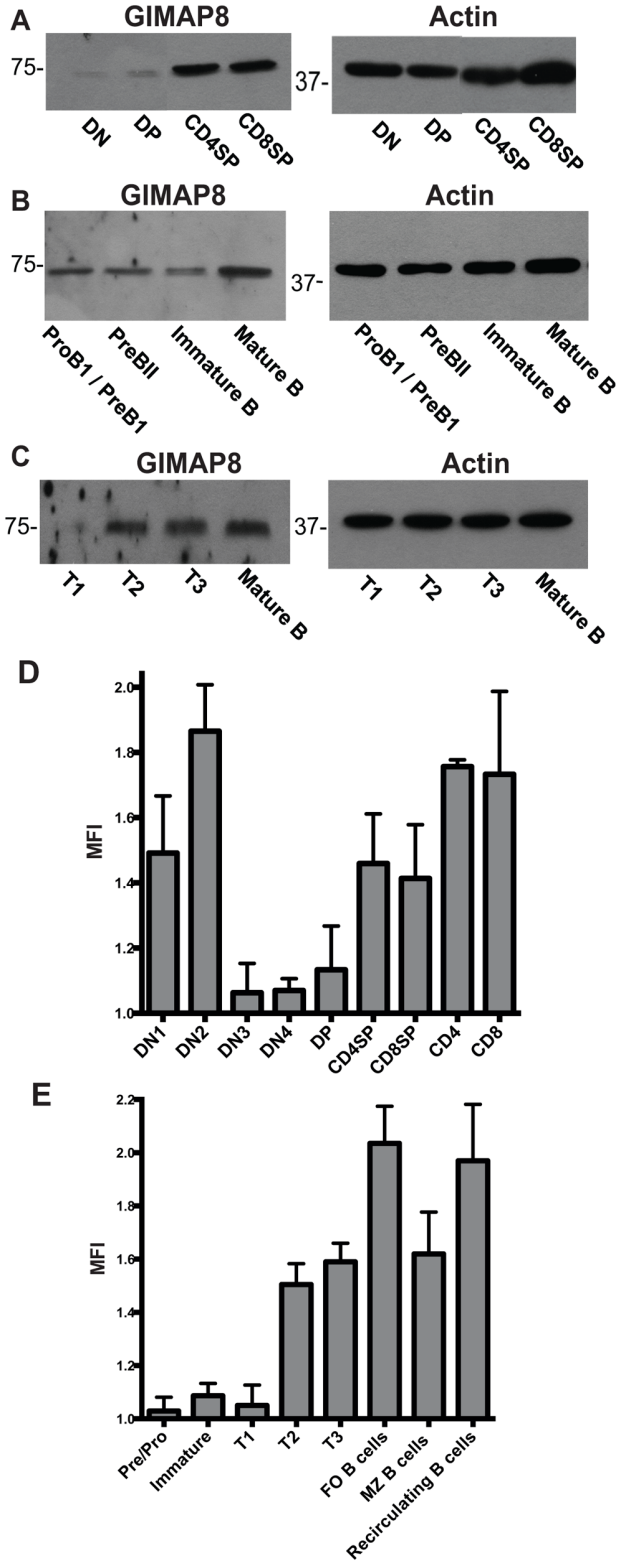
GIMAP8 protein expression during T and B lymphocyte development. Thymocyte, bone marrow, and splenic subpopulations of developing lymphocytes were sorted and subjected to Western blotting to determine GIMAP8 expression during T cell development (A), B cell subsets in the bone marrow (B), and the spleen (C). Actin was used as a loading control. Intracytoplasmic flow cytometry was performed on developing T (D) and B (E) lymphocytes. All cells were stained with extracellular markers to define distinct subpopulations prior to intracytoplasmic staining for GIMAP8 with mAb MAC 418. Results are representative of two independent experiments. Median levels of fluorescence were determined by dividing levels of fluorescence in wild type cells by the average median fluorecence for the same cell type from GIMAP8-deficient animals. Results show mean levels of fluorescence for 3 individual animals ± S.D.

In previous work we have looked at expression of GIMAPs 1, 4, 5, 6 and 9 proteins in subsets of mature and developing B and T lymphocytes by Western blotting. Up to now, the pattern of GIMAP8 expression has only been shown at the mRNA level [39]. We developed novel mAbs recognising mouse GIMAP8 (MAC443 and MAC418) to look at GIMAP8 protein expression in different lymphocyte subsets. Double-negative (DN), double-positive (DP), CD4^+^ single-positive (SP) and CD8^+^ SP thymocytes were purified by FACS, lysed and subjected to Western blotting to detect GIMAP8 expression. This was relatively weak in the earliest stages of T cell development (DN and DP thymocytes) and strongest in SP CD4^+^ and CD8^+^ thymocytes (Figure 1A). We also used Western blotting to examine the expression of GIMAP8 during B cell developmental stages. B lymphocytes showed an expression pattern for GIMAP8 similar to that of the T lineage with expression increasing as cells mature in spleen (Figure 1B) and bone marrow (Figure 1C). To extend these observations, we analysed intracellular GIMAP8 expression by flow cytometry. This enabled us to examine rarer populations of DN thymocytes without the need for purification. MAC418 anti-GIMAP8 performed better in intra-cytoplasmic staining than MAC443. Our data (using MAC418) confirmed the results from Western blotting but also revealed that GIMAP8 is highly expressed in the very earliest stages of T cell development (DN1 and DN2 subsets), diminishing thereafter as cells progress through the β-selection point (Figure 1D). We also showed that GIMAP8 is not expressed until the T2 stage of splenic B cell development and is expressed maximally in the most mature B cell subsets (follicular, and recirculating B cells) with slightly lower expression in marginal zone B cells (Figure 1E). This mirrored the expression seen in these subsets by Western blot (Figures 1B and 1C). This pattern of increased expression in mature lymphocytes is also seen in other GIMAPs (with the exception of GIMAP1 which shows a more uniform expression) [19]. It suggests that GIMAP8 may be important for the development and function of mature B and T lymphocytes.

We were interested to address the intracellular localisation of GIMAP8. Fractionation of lymphocytes into cytosolic/soluble (100 000g supernatant) and membrane-associated (100 000g pellet) fractions showed the expected distribution of the transmembrane-anchor containing GIMAPs 1 and 5 in the membrane-associated fraction. GIMAP8, together with GIMAPs 4, 6, 7 and 9, was predominantly localised to the cytosolic/soluble fraction (Figure S1), although it (together with GIMAPs 6 and 9) showed low levels of membrane-association. Thus GIMAP8 is predominantly a cytosolic protein but may under certain conditions become membrane-associated, as we have previously demonstrated for GIMAP6 [33].

### Generation of GIMAP8-deficient mice

**Figure 2:**
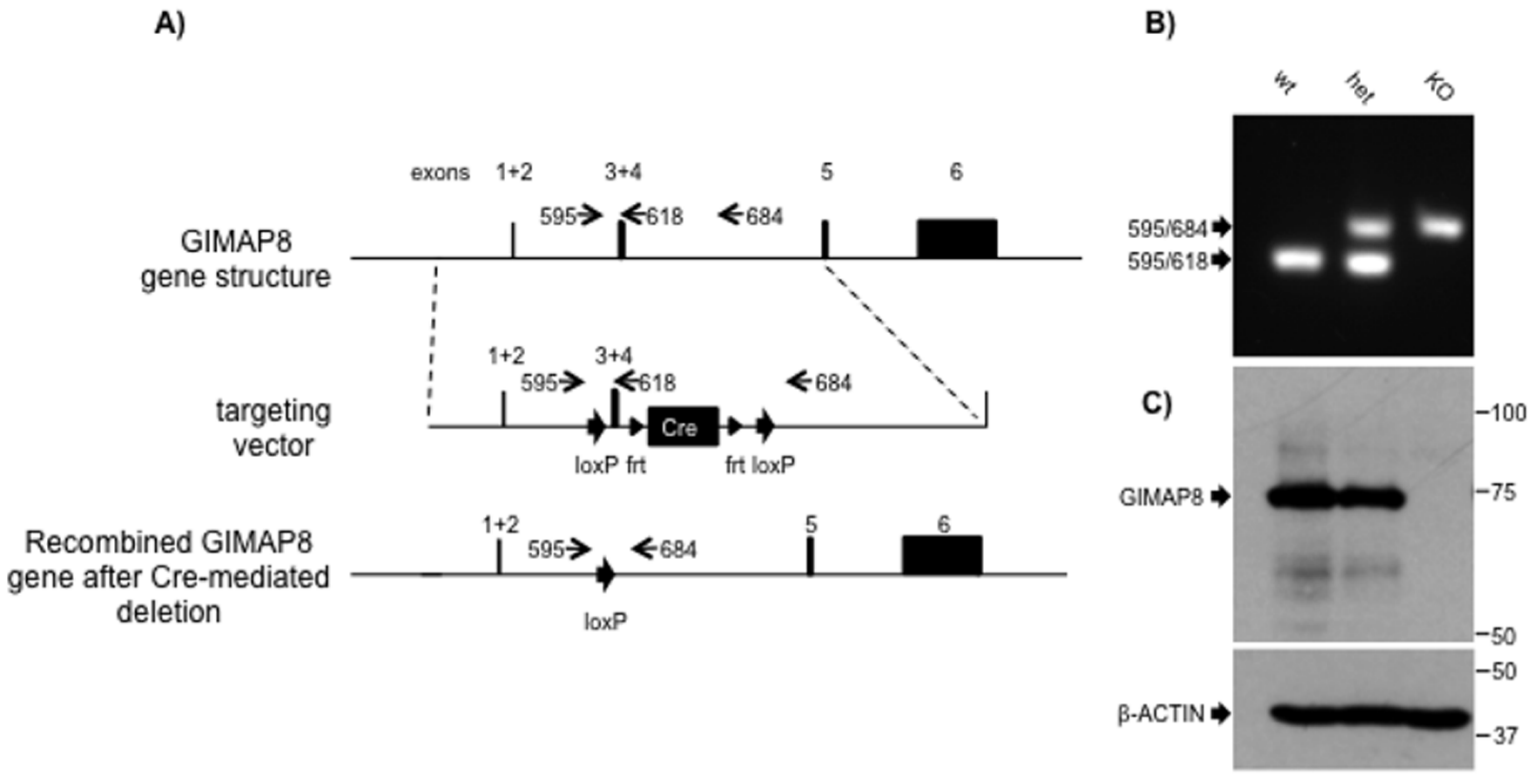
Targeted deletion of exons three and four of the GIMAP8 gene disrupts production of GIMAP8 protein. (A) Summary of the structure of the mouse GIMAP8 gene, the derived targeting vector and the recombined GIMAP8 gene after homologous recombination. Note that for both exons 1 and 2 and exons 3 and 4, the gene structure is represented by single solid blocks as the intervening introns are too small to show at the same scale. Numbered arrows indicate the approximate location of oligonucleotide primers used to identify wild-type and recombined alleles. (B) Agarose gel analysis of PCR products derived using the indicated primer pairs from wild type, heterozygous and homozygous GIMAP8-targeted mice. (C) Upper panel – Western blot of GIMAP8 expression in splenocytes from wild type, heterozygous and homozygous GIMAP8-targeted mice using rat anti-mouse monoclonal antibody MAC 443; Lower panel – the GIMAP8 blot re-probed with an anti β-actin antibody. In both panels, the mobilities of molecular weight standards resolved on the same gel are indicated.

To understand the role that GIMAP8 might be playing in lymphocyte biology we generated and analysed GIMAP8-deficient mice. A targeting construct for mouse *GIMAP8* was designed to delete exons 3 and 4 of the *GIMAP8* gene. Although this recombination event would be predicted to leave significant amounts of the *GIMAP8* gene intact, any nuclear RNA splicing from exons 2 to 5 was expected to result in an out-of-frame fusion and hence fail to produce significant amounts of protein. The resulting mice were backcrossed for six generations on to the C57BL/6 background. Cell lysates from the spleens of wild type (WT), heterozygote, and knockout (KO) mice were subjected to Western blot analysis using anti-GIMAP8 (MAC 418) mAb as a probe. Results show the presence of GIMAP8 protein in WT and heterozygous splenocytes and its absence from *GIMAP8^−/−^* splenocytes (Figure 2C), confirming deletion of GIMAP8 protein. Interbreeding of heterozygous mice produced offspring at the expected Mendelian ratio. *GIMAP8^−/−^* mice were fertile and showed no signs of physiological defects.

### Normal T lymphocyte development in GIMAP8-deficient mice

**Figure 3:**
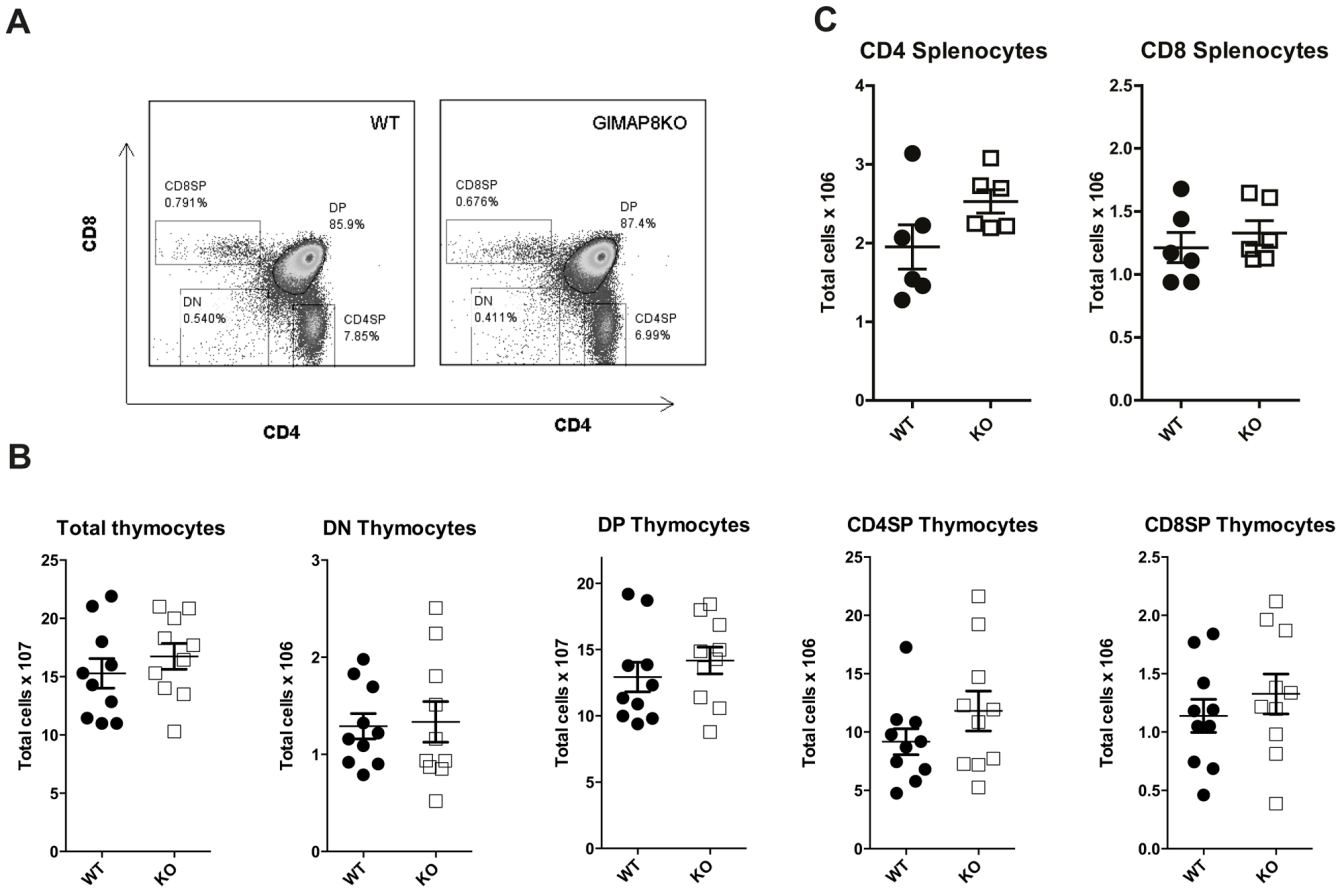
Thymocyte development in GIMAP8-deficient mice. (A) Representative flow cytometry plots of thymocytes from *GIMAP8^−/−^* mice and littermate controls stained for CD4 and CD8. (B) Numbers of thymocyte subsets are shown where each dot is representing a single mouse. (C) Numbers of CD4 and CD8 splenocytes are shown where each dot is representing a single mouse.

We have previously shown that deletion of GIMAP1 results in a profound deficit of mature lymphocytes in the periphery [19]. In the T cell lineage, this defect becomes apparent in the thymus, where there is a significant reduction in the number of mature SP thymocytes. To determine whether GIMAP8 also plays a role in T cell development we examined the thymus, spleen, and lymph nodes of GIMAP8-deficient and WT mice. As shown in Figure 3A, there were no differences in the proportions of DN, DP and SP thymocytes. No differences were found when each subset was enumerated (Figure 3B), indicative of normal T cell development in the thymus. We also assessed CD4 and CD8 lymphocytes in the spleen (Figure 3C) of GIMAP8-deficient mice. The numbers of neither subset were affected by the absence of GIMAP8 (Figure 3C). The numbers of naïve (CD62L^hi^CD44^lo^) and memory (CD62L^l^°CD44^hi^) CD4 and CD8 cells were also unaffected by the absence of GIMAP8 (Figure S2).

### Reduction in recirculating B cells in bone marrow of GIMAP8-deficient mice

**Figure 4:**
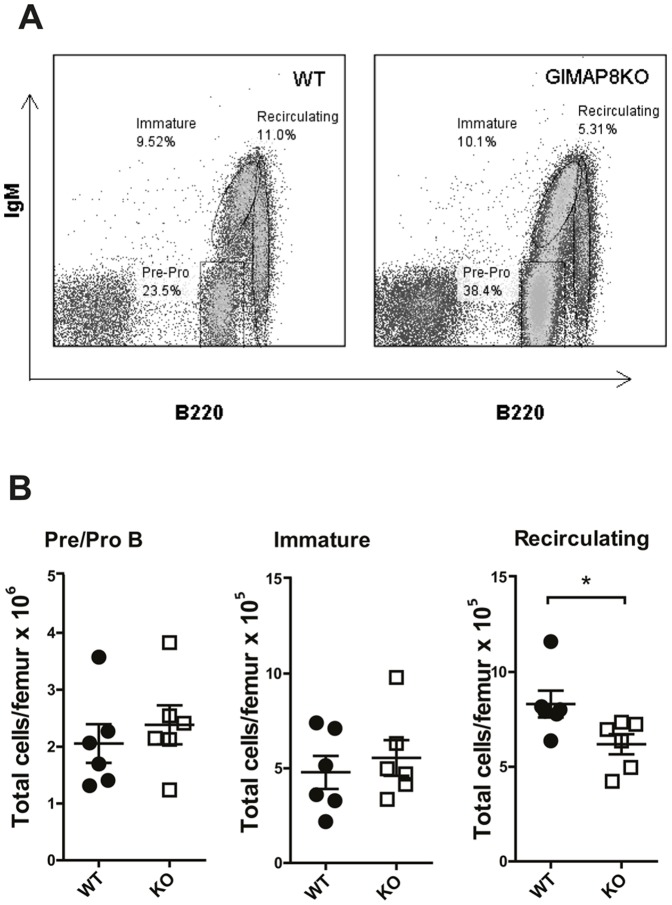
Bone marrow cell analysis. Bone marrow cells from *GIMAP8^−/−^* mice and littermate controls were stained for flow cytometric analysis to identify B-lineage subsets. (A) Representative flow cytometry plots for each genotype. The numbers given inside the component panels in (A) are the percentages of different bone marrow B cell populations. (B) Numbers of B-lineage subsets in BM are shown where each dot represents a single mouse.*P <0.05 (unpaired 2-tailed Student's t test).

Next, we examined B cell subsets in the bone marrow of GIMAP8-deficient mice. Cells were enumerated and then stained to determine numbers of pre/pro, immature, and recirculating B cells. As shown in Figure 4A, deletion of GIMAP8 affected the proportion of recirculating B cells in the bone marrow. This was also evident when recirculating B cells were enumerated (Figure 4B). *GIMAP8^−/−^* mice had around 25% fewer recirculating B cells than their WT counterparts. In contrast, numbers of pre/pro and immature B cells were unaffected (Figure 4B). This suggests that B cell development in the bone marrow is not dependent upon GIMAP8 but that mature recirculating B cells are affected when GIMAP8 is deleted. Previously we found that deletion of GIMAP1 also had little effect on B cell development within the bone marrow but caused significant problems during the transitional stages of B cell development within the spleen [19]. Thus, we examined immature and mature B cell subsets in the spleen of GIMAP8-deficient mice.

### Normal B cell development in the spleen of GIMAP8-deficient mice

**Figure 5:**
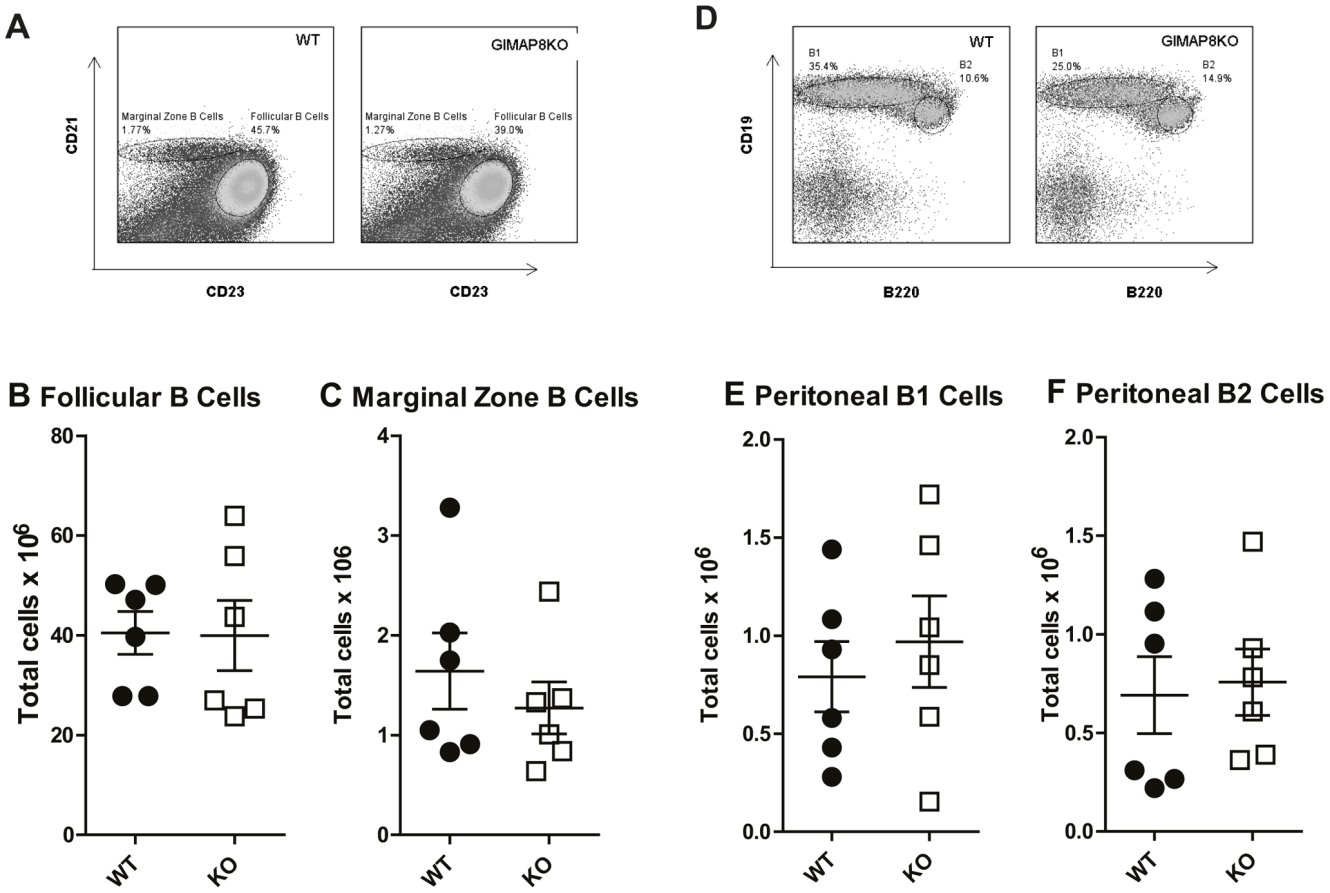
Splenic and peritoneal B cell analysis. (A) Representative flow cytometry plots of splenic cells for each genotype. The numbers given inside the component panels in A and D are the percentages of lymphocyte events contained within the lymphocyte gate. Numbers of B-lineage subsets in spleen and peritoneum are shown where each dot represents a single mouse. Numbers of mature B cell subsets in spleen (Figures B & C). Representative flow cytometry plots of peritoneal cells for each genotype (D). Numbers of B-lineage subsets in spleen and peritoneum are shown where each dot represents a single mouse (Figures E & F).

During B cell development, immature B cells formed in the bone marrow migrate to the spleen where they undergo further differentiation into transitional immature B cells prior to their final maturation [40]. The expression of CD93 (AA4.1) is used in addition to CD23 and IgM to divide transitional B cells into three non-proliferative B cells subsets – T1, T2, and T3. Transitional B cells develop into mature follicular B cells (FO) and marginal zone B cells (MZ). We examined proportions and numbers of the T1, T2, and T3 transitional subsets alongside FO and MZ B cells in WT and GIMAP8-deficient mice. We found no effect of GIMAP8 deletion on splenic B cell subsets with normal numbers of transitional (data not shown), FO and MZ B cells being observed (Figure 5). Thus, it appears that GIMAP8 is not required for B cell development or survival within the spleen. We also looked for the presence of mature B cells in the lymph nodes of GIMAP8-deficient mice. Similar to splenic B cells, GIMAP8 deficiency had no impact on the number of B cells within lymph nodes (Figure S3). Both GIMAP1 and GIMAP5 have been shown to affect peritoneal B cells. However, as shown in Figures 5D-F, absence of GIMAP8 has no effect on the number of B1a, B1b, or B2 cells resident within the peritoneum.

### *In vitro* survival of mature peripheral B and T cells from GIMAP8-deficient mice

**Figure 6:**
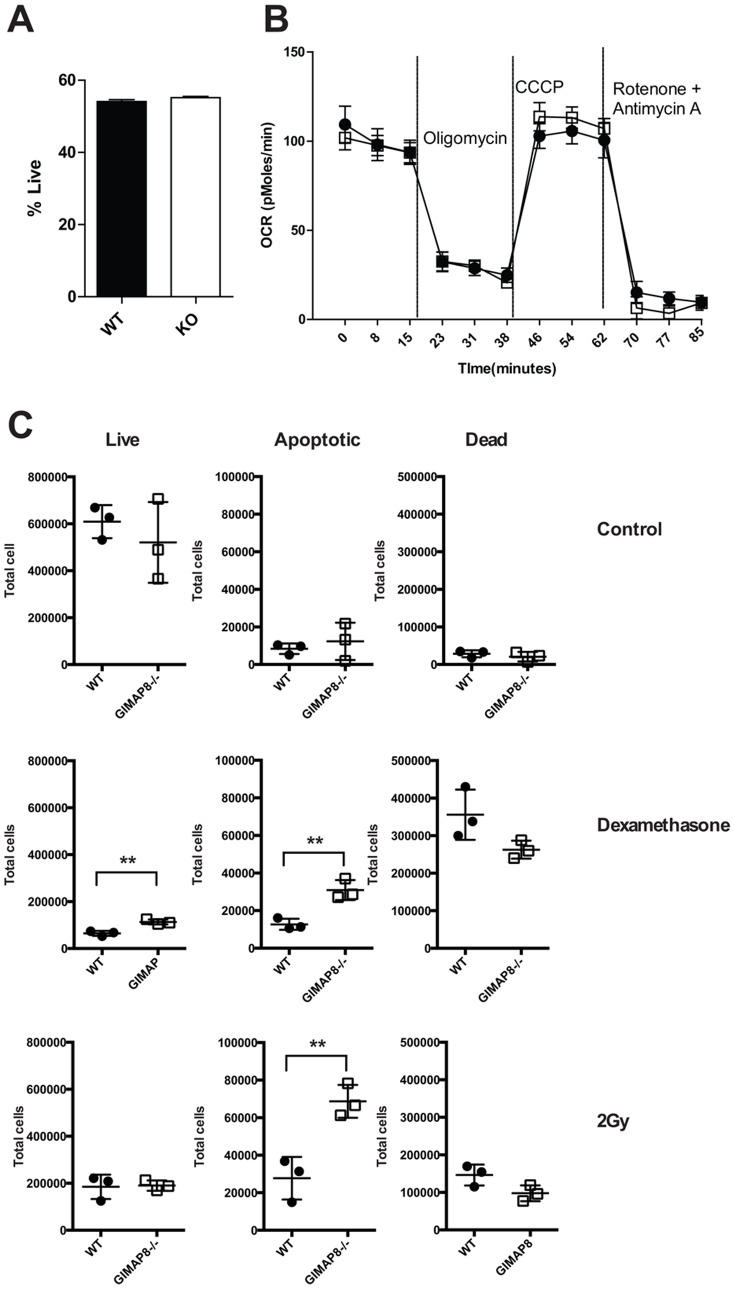
B cell survival and respiration *ex vivo*. (A) the mean number (± SD) of live cells as a percentage of the original number of cells plated for three individual mice. (B) Mean (± SD) OCR for triplicate wells containing purified splenic B cells from WT WT (▪) and GIMAP8-and deficient mice (□) mice following injection of oligomycin, CCCP, and antimycin A plus rotenone and is representative of two independent experiments. (C) T cells from either WT (•) and GIMAP8-and deficient mice (□) were incubated in complete medium (control), dexamethasone, or following gamma irradiation for 8 hr. The number of live (Annexin V- and DAPI-), apoptotic (Annexin V+ and DAPI-), and dead (AnnexinV+ or - and DAPI+) cells were enumerated by flow cytometry. Figure 6C shows the average count for duplicate samples from three mice and is representative of two independent experiments. *p<0.05, ** P<0.005 (unpaired 2-tailed Student's t test).

To further examine the reduction of mature recirculating B cells in the bone marrow of GIMAP8-deficient mice we looked at the ability of mature B cells to survive *ex vivo*. B cells were purified from the spleen of WT or *GIMAP8^−/−^* mice and cultured *in vitro* for 24 hours. Cell death was measured on the basis of a reduced cell size (as measured by forward scatter) and uptake of 7AAD (indicative of increased cell membrane permeability). As shown in Figure 6A, *GIMAP8^−/−^* and WT B cells showed similar levels of cell death. We also looked at the respiratory capacity of WT and *GIMAP8^−/−^* B cells using a Seahorse Extracellular Flux Analyser to measure oxygen consumption rate (OCR) and spare respiratory capacity (SRC). Defects in SRC (the maximal respiratory capacity) could account for the inability of mature cells to survive when they transit into the bone marrow. However, GIMAP8-deficient B cells had similar OCR and SRC to WT cells indicating that a defect in metabolism was unlikely to account for the paucity of recirculating B-cells within the bone marrow niche (Fig. 6B).

Previous studies by ourselves and others have shown that mature T cells from mice and rats deficient in GIMAP4 have perturbed apoptosis [25] [24]. We assayed apoptosis induction in mature T cells from wild type and GIMAP8-deficient mice. Mature CD4 and CD8 T cells were purified by negative depletion and cultured for 8 hours. Cell death was induced by either dexamethasone or gamma irradiation and assessed by Annexin V binding and DAPI uptake. As shown in Figure 6C, GIMAP8-deficient T cells show a decrease in the total number of dead cells (DAPI^+^) after apoptosis induction and a concomitant increase in the number of apoptotic cells (Annexin V^+^ and DAPI*^−^*) compared to wild type T cells.

### The *in vivo* immune reponses to a T-dependent antigen in the absence of GIMAP8

**Figure 7:**
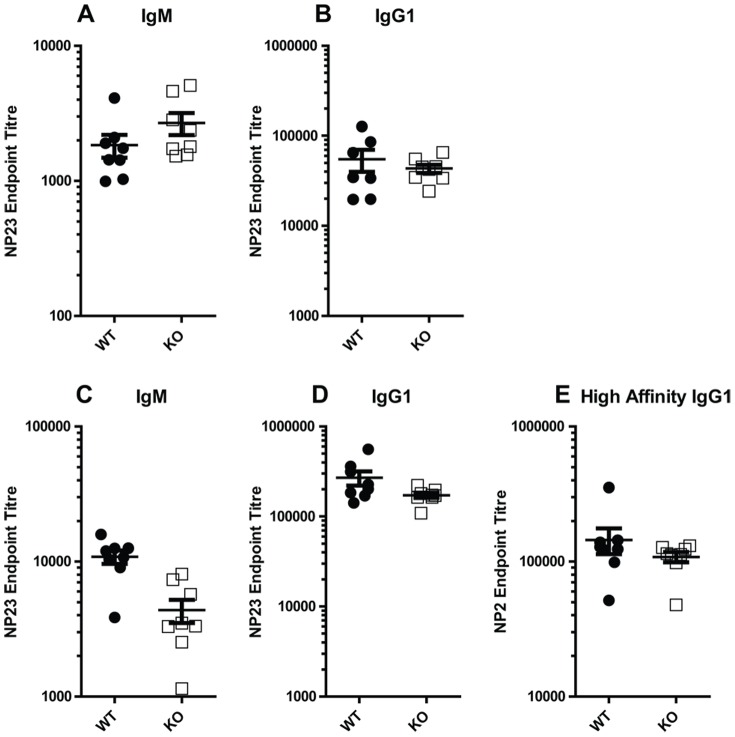
T cell-dependent immune responses in *GIMAP8^−/−^* mice. Day 14 titers of anti-NP_23_ IgM (A) and anti-NP_23_ IgG_1_ (B). Five weeks after primary immunization, mice were immunized with NP-KLH to examine secondary responses. Titers of anti-NP_23_ IgM (C), anti-NP_23_ IgG_1_ (D), and high affinity anti-NP_2_ IgG1 7 days after secondary immunization. Each dot represents a single mouse.

To determine if a lack of GIMAP8 affected the function of lymphocytes, we immunised mice with a thymus-dependent antigen and measured the serum levels of antigen-specific IgM and IgG1 14 days later. This allowed us simultaneously to detect any possible defects in the immune cells involved in T-dependent antigenic responses and any defects in either the antigen presenting cells, T or B lymphocytes which could have affected the titres and class of antibodies produced. As shown in Figures 7A-B, serum levels of both NP-specific IgM and IgG1 did not differ between WT and *GIMAP8^−/−^* mice. Mice were challenged with a second dose of NP-KLH and both high and low affinity antibodies to the antigen measured. As shown in Figures 7C–E, lack of GIMAP8 has no effect on levels of low and high affinity IgG1, indicative of normal affinity maturation and memory cell formation and function. Following the secondary response there is a small decrease in the levels of NP-specific IgM (Figure 7C).

## Discussion

To date, very little is known about the role GIMAP8 might play in lymphocyte biology. Previous work has shown that GIMAP8 mRNA is expressed highly in thymus tissue and is weakly detectable in heart, skin, and lung [6], [17], [28]. Work from our group has also shown that GIMAP8 mRNA is expressed most strongly in mature lymphocytes and very weakly in the early DN population of thymocytes [39]. Overexpression of GFP-tagged GIMAP8 in transfected CHO-K1 cells has shown that GIMAP8 localizes to the ER, Golgi, and mitochondria [17]. However, localization of native GIMAP8 has not yet been elucidated. The unique structure of GIMAP8, namely its three GTP binding domains, suggested that it might play a role that differs from that of other GIMAP family members. GTPases containing more than one GTP binding domain are rare, yet appear to be a consistent feature of the vertebrate GIMAP cluster (Figure S4). The bacterial protein EngA contains two GTP binding domains, which are thought to make distinct contributions to the protein's function [41]. EngA orthologs are present in all bacterial genomes and *Arabidopsis*. Although the exact function of these proteins has not been elucidated, studies have revealed that the *Escherichia coli* EngA homolog, Der, and *Neisseria gonorrhoeae* EngA are essential for cell viability [42]–[44]. Nucleotide binding to the first GTPase domain of EngA triggers a dramatic conformational change in the protein which exposes a positively charged surface over the central domain and the second GTPase domain. This is thought to confer ribosome binding properties to the enzyme. In addition, the two GTP binding sites in EngA are not equivalent. The more C-terminal site has an exceptionally slow intrinsic GDP release rate [44]. The conserved sequence differences between the different GTP binding domains of GIMAP8 suggest that similar subtle biochemical mechanisms may also be important for the overall function of GIMAP8.

GIMAP8-deficient mice were born at expected Mendelian ratios, were fertile, and showed no obvious morphological defects. T and B cell development was normal and the expected numbers of mature peripheral T and B cells were detected in spleen, lymph nodes, and peritoneum. The reduction in the proportion and numbers of recirculating B cells within the bone marrow environment is an intriguing finding. Since deletion of GIMAP8 had no effect on splenic, peritoneal and lymph node B cells it suggests that there is something about the bone marrow niche that means that B cells depend upon GIMAP8 either intrinsically or that other cell types within the bone marrow niche must express GIMAP8 to assist recirculating B cells. The most likely possibilities are that absence of GIMAP8 affects migration and/or retention of mature B cells within the bone marrow or that GIMAP8-deficient B cells have a survival defect within the bone marrow that is not manifested in other organs. In an attempt to address the possibility of a survival defect, we investigated the ability of mature B cells to survive *ex vivo* when cultured in complete medium. Lack of GIMAP8 had no effect on the number of live cells remaining after 24hr culture. This result does not completely rule out an inherent survival defect in GIMAP8-deficient cells but suggests that any such defect may only be manifested in specific tissue environments which are not mimicked *in vitro*. We also looked at the respiration of B cells from GIMAP8-deficient mice and their SRC but no abnormalities were detected. A defect in SRC might manifest itself in environments where increased energy demands are placed on the cell. Foxo3-deficient mice also have a reduction in the number of recirculating B cells in the bone marrow but normal distribution of B cell subsets within the spleen [45]. It is postulated that in Foxo3-deficient mice this might be due to a reduction in expression of sphingosine-1-phosphate receptor, which contributes to the egress of lymphocytes from peripheral lymphoid organs [46]. However, Foxo3-deficient mice also have a defect in the number of B2 cells in the peritoneum that is not seen in GIMAP8-deficient mice. Additionally, GIMAP5-deficient T cells show a progressive loss in Foxo1, Foxo3, and Foxo4 expression which leads to a reduction in the number of regulatory T cells and loss of immunological tolerance. Alternatively, GIMAP8 may be involved in responses to the chemokine Migration Inhibition Factor (MIF) [47]. MIF is important for survival of recirculating B cells within the bone marrow. In the absence of MIF, there is a normal distribution of B cell subpopulations within the spleen but a marked reduction in the numbers of recirculating B cells in the bone marrow. MIF is thought to be produced by the dendritic cells found in the perivascular clusters within bone marrow niches where it supplies a crucial survival signal to the recirculating B cell population. Although our phenotype within the bone marrow is not as dramatic as that seen in the MIF knockouts, it suggests that GIMAP8 could affect the B cell's ability to respond appropriately to MIF or MIF production within perivascular clusters.

Curiously, mature GIMAP8-deficient T cells showed an apparent delay in apoptotic cell death in response to dexamethasone and gamma-irradiation. This *in vitro* phenomenon has previously been reported for GIMAP4-deficient T cells from both mice and rats [24] [25] and it was suggested that it may reflect an *in vivo* role for GIMAP4 in accelerating the clearance of dying cells during lymphocyte development (e.g. in T cell negative selection) or during the contraction phase of immune responses [48] [25]. As with GIMAP4, our results suggest that, although the immune system can develop and function in the absence of GIMAP8, apoptosis pathways are perturbed when cells lack GIMAP8. The real function of GIMAP8 in regulating apoptosis in mature cells may be masked by redundancy within the GIMAP family. In spite of this finding, GIMAP8 deficiency had no effect on T-dependent antibody responses. The role of GIMAPs in immune responses has not been addressed extensively. GIMAP1 and GIMAP5 deficient animals have very few peripheral mature lymphocytes and the T cells in the periphery show evidence of homeostatic proliferation, thus making comparison with WT T cells difficult to interpret. *GIMAP5^sphinx/sphinx^* mice (a recessive *N*-ethyl-N-nitrosourea-induced mutation in *GIMAP5* which results in a GIMAP5-deficiency) show defects in both T and B cell antigen receptor-induced proliferation and are unable to mount T-dependent responses following immunisation with a T-dependent antigen [23]. In addition, data from our own laboratory using an inducible knockdown system suggest that GIMAP1 is important for both T and B cell proliferation (L.Webb and G.Butcher unpublished data). However, GIMAP8 deficiency had no effect on the ability of T and B cells to respond to the T-dependent antigen, NP-KLH, *in vivo*. The normal levels of NP-specific IgG1 antibodies of both low and high affinity are indicative of normal T and B cell function and also suggest that there are no defects in GIMAP8-deficient antigen presenting cells.

In conclusion, we find that GIMAP8 is expressed in T and B lymphocytes and its expression increases as cells mature and migrate into the periphery. However, we find that most lymphocytes can develop and survive in the absence of GIMAP8, suggesting that it is redundant for lymphocyte development. We also find that T-dependent antigen responses are normal in the absence of GIMAP8. These results are surprising given the unique structure of the GIMAP8 protein. They suggest that GIMAP8 function may often be compensated for by other proteins and that its unique function in lymphocyte biology may only be revealed under as yet unknown conditions of specific immunological or pathogenic stress.

## Materials and Methods

### Mice

Mice were bred and maintained in specific pathogen-free conditions at The Babraham Institute. Husbandry and experimentation complied with existing European Union and United Kingdom Home Office legislation and local standards and are approved by the Babraham Institute Animal Welfare, Ethics, and Experimentation Committee. Male and female mice were used at 8 to 16 weeks of age for each set of experiments.

### Generation of GIMAP8-deficient mice

Recombineering was used to develop a targeting vector for GIMAP8 using BAC RP23-97A20 from the C57BL/6 RPCI-23 BAC library as the starting material [49]. A loxP site was inserted upstream of exon 3 of the *GIMAP8* gene (exon nomenclature according to Ensembl mouse genome assembly GRCm38) and an frt1-flanked neo cassette with a 3′-flanking loxP site was inserted downstream of exon 4 (see Fig. 2A). The construct was electroporated into BRUCE-4 C57BL/6 ES cells and transformants were selected using G418. Cells carrying a correctly targeted *GIMAP8* locus were identified by Southern blotting using probes flanking the 5′- and 3′- ends of the targeting vector. Mice transmitting the correctly targeted *GIMAP8* allele to offspring were derived as described previously [19], and identified by PCR analysis across the 5′-loxP site using primers 5′-GGAGATGAGGCTTAGCTTGC-3′ and 5′-GCTTGTAATCACGGAGCAGC-3′. These mice were bred to mice carrying a flpE transgene to delete the neomycin resistance cassette, and then to Protamine-Cre transgenic mice to mediate germline recombination between the two loxP sites and hence delete GIMAP8 exons 3 and 4 (see Fig. 2A). Mouse genotypes were routinely identified by PCR from “hot-shot” DNA from tail or ear biopsies using a PCR reaction containing three primers: 595 5′-GGAGATGAGGCTTAGCTTGC-3′; 618 5′-AGGTTTACTTGCCCTGTATCC-3′; 684 5′-ACCCTGGCTGGCACTGTTGAG-3′. This PCR gives products of 291 base pairs for the wild-type allele and 347 base pairs for the recombined targeted allele.

### Generation of anti-GIMAP8 monoclonal antibody

Monoclonal rat IgG antibodies raised against truncated rat GIMAP8 (amino acids 463-688 rat; MAC418) or full-length mouse GIMAP8 (MAC443) fused to glutathione-S-transferase were prepared using protocols described previously [33]. MAC418 anti-rat GIMAP8 crossreacts on the mouse homologue.

### Western blotting

Cell samples at a concentration of 2 × 10^8^/ml were incubated in lysis buffer (2% Nonidet P-40, 20mM Tris, 150mM NaCl, and 1mM MgCl_2_ [pH8] supplemented with proteolytic inhibitors) for 30 min at 4°C. Nuclei were removed by centrifugation at 14,000g. An equal volume of Laemmli sample buffer (Bio-Rad) was added to each sample. Ten-microliter samples, equivalent to 1 × 10^6^ cells, were analysed by SDS-PAGE gel electrophoresis and Western blotting as described previously [19] with anti-mouse GIMAP8 mAb MAC443. After staining with anti-GIMAP8 antibody, blots were stripped with Restore Western Blot stripping buffer (Pierce) and stained with monoclonal anti-β-actin (Sigma Aldrich) as a loading control.

### Subcellular fractionation of splenic lymphocytes

Single cell suspensions from mouse spleens were prepared by passage through a 70µM filter. Red cells were lysed as described previously [19] and the remaining lymphocyte fraction homogenised in homogenisation buffer (10 mM acetic acid, 1 mM EDTA, 190 mM sucrose, 10 mM triethanolamine, pH 7.4 with 1:100 Mammalian Protease Inhibitor Cocktail) using a ball-bearing homogeniser as described previously [26]. Post nuclear fractions were prepared by centrifugation at 1000g for 10 minutes at 4°C. An aliquot of supernatant was removed to represent the post-nuclear supernatant and the remainder centrifuged at 100 000 g for 1 h at 4°C in a TLS-55 swing-out rotor (Beckman-Coulter). The supernatant was removed and retained (100,000 g supernatant) and the pellet re-suspended in homogenisation buffer and spun as before. The resulting pellet was re-suspended in homogenisation buffer (100, 000 g pellet) and equal volumes of 2 × complete SDS gel sample buffer (160 mM Tris pH 6.8, 4% SDS,20% glycerol, 5% 200 mM dithiothreitol) added to each fraction. Samples were boiled and then analysed for the distribution of individual GIMAP species by Western blotting using a panel of in-house derived monoclonal antibodies.

### Flow cytometric analysis and enumeration of lymphocytes

Single-cell suspensions from mouse thymus, spleen, lymph nodes (inguinal), and bone marrow (BM) were stained for flow cytometry. Peritoneal cells were obtained by washing the peritoneum with 5 ml of PBS/3 mM EDTA/0.5% BSA. Cell staining was performed using biotinylated or fluorochrome-conjugated antibodies against CD3, CD4, CD5, CD8, CD19, CD21, CD23, CD24, CD25, CD93, T-cell receptor β (TCRβ), B220, immunoglobulin M (IgM), c-kit, Thy1.2, and Qa2, as well as fluorochrome-conjugated streptavidin as a second-stage reagent (eBioscience; BD Biosciences). DN, DP, CD4SP, and CD8SP thymocytes were identified on the basis of CD4, CD8, and TCR expression (DN CD4-CD8-; DP CD4+CD8+; CD4SP CD4+CD8-; CD8SP CD4-CD8+TCRhi). ProB1/PreB1, PreBII, Immature B, and Mature B cells were identified on the basis of IgM, IgD, B220, and c-kit expression (ProB1/PreB1 IgM-B220intCD25+; PreBII IgM-B220intCD25-; Immature B IgM+B220int; Mature B IgM+B220hiIgD+). Splenic T1, T2, T3, Marginal Zone, and Follicular B cells were identified on the basis of CD23, CD21, CD93, and IgM expression (T1 CD93+CD23-IgM+; T2 CD93+CD23+IgM+; T3 CD93+ CD23+IgM-; MZ CD93-CD21hiCD23-; FO CD93-CD21intCD23hi). DN thymocytes were identified by lack of CD4, CD8, B220, γδTCR, NK1.1, Gr1 and CD11b expression and fractionated on the basis of CD44 and CD25 expression (DN1 CD25-CD44+; DN2 CD25+CD44+; DN3 CD25+CD44-; DN4 CD25-CD44-). CD4 and CD8 expression was used to identify CD4 and CD8 splenic T cells. After staining, cells were washed and resuspended and then analysed on an LSRII Flow Cytometer (BD Biosciences). Data analysis was performed using FlowJo (TreeStar Inc) software. Cells were enumerated by an automated cell counter (CASY; Schärfe).

### Fluorescence-activated cell sorting

Single-cell suspensions of thymocytes, BM cells, or splenocytes were stained as described above and cell populations were sorted using a FACSAria (BD Biosciences). Purities were between 90% and 99.9%.

### Intracellular staining for GIMAP8

Lymphocytes isolated from spleen, bone marrow, lymph nodes, and thymus of C57BL/6 mice were stained for surface markers described above. Cells were then simultaneously fixed and permeabilized with BD Cytofix/Cytoperm, and washed in FACS buffer (PBS with 0.5% BSA and 0.01% sodium azide) containing 0.03% saponin (permeabilization buffer) before incubation with anti-GIMAP8 mAb (MAC418) for 1 hr at 4°C. Next, cells were washed in permeabilization buffer and then stained with FITC-anti-rat IgG in the presence of 10% normal mouse serum for 1 hr at 4°C. Cells were then washed and analysed on a flow cytometer (LSRII) and data were analysed using Flowjo software (Tree Star). Cells from GIMAP8-deficient mice were used to determine background staining.

### Immunizations

Mice were immunized i.p. with 100 µg of (4-hydroxy-3-nitrophenyl)acetyl (NP)_19_–keyhole limpet hemocyanin (KLH; Biosearch Technologies, Novato, CA) adsorbed to alum. Mice were bled at the indicated time points before boosting with soluble antigen and antibody titres determined as previously described [50].

### Cell survival assay

B lymphocytes were purified from lymph nodes by depletion of CD43-expressing cells using AutoMACS (Miltenyi, Millipore). Briefly, cells were incubated with biotinylated anti-CD43 mAb for 30 min at 4°C and then washed in PBS containing 1% BSA. The cells were then incubated at 4°C for 30 min with anti-biotin microbeads (MACS). Cells were washed and then depleted of CD43-expressing cells using an AutoMACS machine according to the manufacturer's instructions. Cells were>95% CD19+ve and>95% viable as determined by flow cytometry. Cells were then incubated for 24 hr in complete medium (IMDM supplemented with 10% (v/v) heat-inactivated FCS, 1% (v/v) penicillin/streptomycin (5000 µ/ml) and 50µM β-mercaptoethanol). Cells were then counted using a CASY counter, stained with 7AAD (Sigma) and run on a LSRII flow cytometer; results were analysed using FlowJo (TreeStar).

### Apoptosis assay

CD4 and CD8 T cells were enriched from spleen and lymph nodes of 8–16 week old wild type and *GIMAP8^−/−^* mice by depletion of non-T cells using biotinylated antibodies against CD19, IgD, CD11b, CD11c, NK1.1, Gr1, and γδTCR followed by anti-biotin Macs beads and AutoMACs as described above. Cells were>90% CD4 or CD8 and cultured for 8 hr in complete medium with either 1 µM dexamethasone or following gamma-irradiation (2 Grays). Apoptosis was assessed by Annexin V binding and DAPI uptake and cells enumerated using FlowCount beads. Cells were analysed on a Fortessa Flow Cytometer (BD Biosciences) and data analysed using FlowJo (TreeStar).

### Seahorse assay

We used the Seahorse Extracellular Flux (XF) 96 Analyzer (Seahorse Bioscience, Inc, North Billerica, MA, USA), to measure the oxygen consumption rate (OCR), an indicator of mitochondrial respiration, in real-time in live intact B lymphocytes as previously described [51]. The effect of oligomycin, CCCP, antimycin A, and rotenone (all from Sigma Aldrich, UK) on B cell OCR was measured to assess spare respiratory capacity (SRC).

## Supporting Information

Figure S1 Distribution of GIMAPs between soluble and membrane-associated fractions in lysates from splenic lymphocytes. Proteins derived from post-nuclear (PNS), soluble (100 000g supernatant), and membrane-associated (100 000 g pellet) fractions derived from splenic lymphocytes were resolved by SDS-PAGE (usually approximately 2 × 10^6^ cell equivalents/lane but 4 × 10^6^ for GIMAP7 gel)) and Western blotted for the distribution of individual GIMAP proteins using in-house derived rat monoclonal antibodies MAC420 (anti-GIMAP1), MAC417 (anti-GIMAP4), MAC421 (anti-GIMAP5), MAC436 (anti-GIMAP6), MAC448 (anti-GIMAP7), MAC443 (anti-GIMAP8) and MAC433 (anti-GIMAP9), followed by horseradish-peroxidase conjugated goat anti-rat IgG, developed with Immobilon Western HRP substrate (MILLIPORE), and imaged using a G:Box (Syngene).
(TIF)

Figure S2 Proprtion of naïve T cells in periphery. Splenocytes from *GIMAP8^−/−^* mice and littermate controls were stained for CD4, CD8, CD44, and CD62L. Plots show proportion of naïve cells for individual mice (• indicates WT, n  =  3, □ indicates *GIMAP8^−/−^*, n  =  3).
(TIF)

Figure S3 Numbers of B cells in lymph nodes. Cells from *GIMAP8^−/−^* mice and littermate controls were counted and stained for B220. Plots show number of cells for individual mice (• indicates WT, n  =  8, □ indicates *GIMAP8^−/−^*, n  =  7).
(TIF)

Figure S4 Alignment of GIMAP8 from various vertebrate classes. GIMAP8 protein sequences were aligned using ClustalW. The sequences had NCBI accession numbers: human (*Homo sapiens*) NP_783161.1; mouse (*Mus musculus*) NP_997651.1; lizard (*Anolis carolinensis*) XP_008106400.1; guppy (*Poecilia reticulate*) XP_008401341.1. The chicken (*Gallus gallus*) cDNA sequence was derived in-house by reverse transcriptase PCR from the DT40 cell line and then *ab initio* translated into protein. Sites at which all five proteins have identical or similar amino-acids are highlighted in red, four in blue and three in grey. G boxes are underlined in black.
(TIF)
