# Supplementary figures and images for: Generation and Characterisation of Mice Deficient in the Multi-GTPase Domain Containing Protein, GIMAP8
Source: PLoS One. 2014 Oct 17;9(10):e110294. doi: 10.1371/journal.pone.0110294 (PMC4201521; doi:10.1371/journal.pone.0110294)

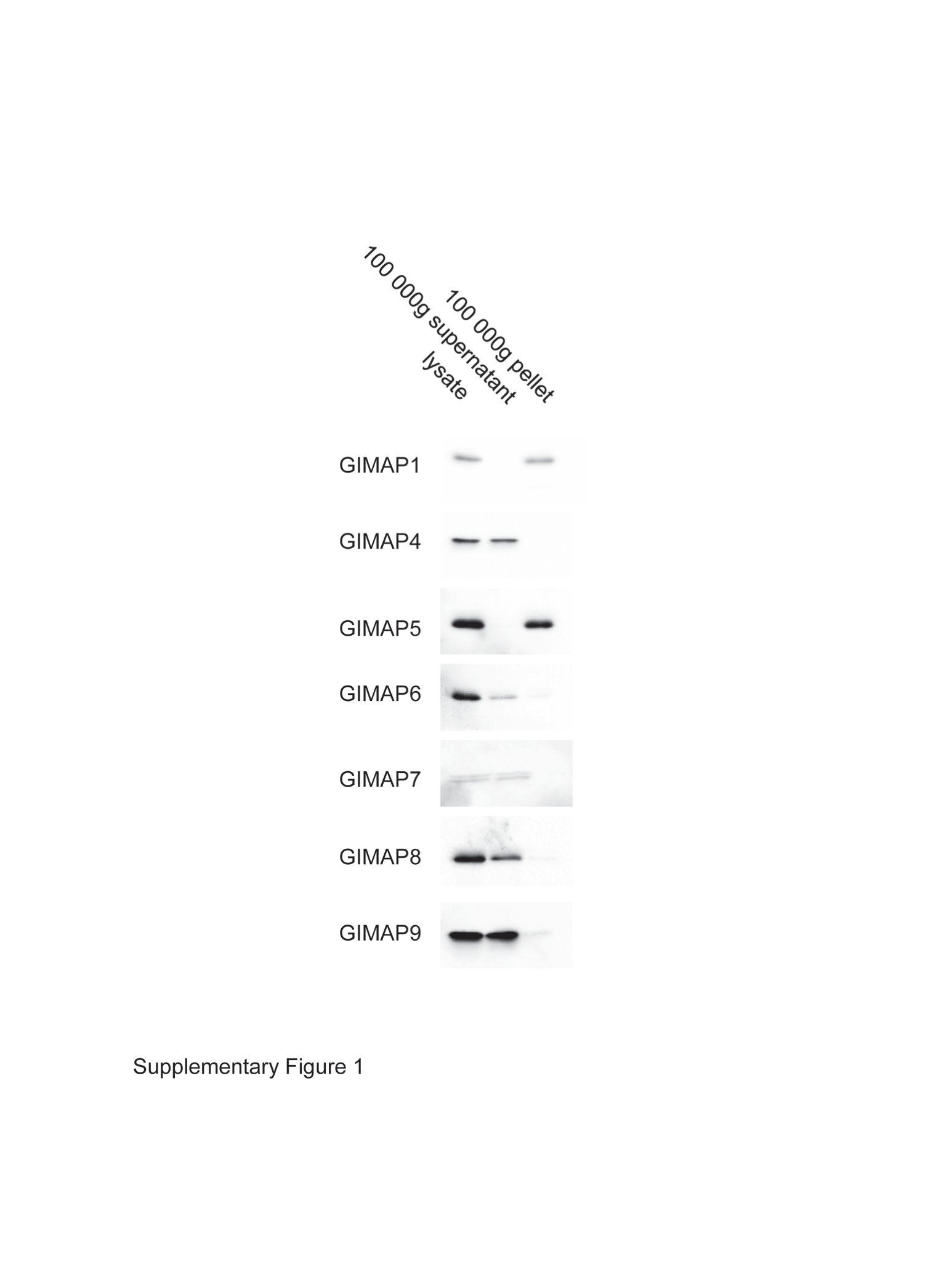

Supplement: Figure S1 — Distribution of GIMAPs between soluble and membrane-associated fractions in lysates from splenic lymphocytes. Proteins derived from post-nuclear (PNS), soluble (100 000g supernatant), and membrane-associated (100 000 g pellet) fractions derived from splenic lymphocytes were resolved by SDS-PAGE (usually approximately 2 × 10^6^ cell equivalents/lane but 4 × 10^6^ for GIMAP7 gel)) and Western blotted for the distribution of individual GIMAP proteins using in-house derived rat monoclonal antibodies MAC420 (anti-GIMAP1), MAC417 (anti-GIMAP4), MAC421 (anti-GIMAP5), MAC436 (anti-GIMAP6), MAC448 (anti-GIMAP7), MAC443 (anti-GIMAP8) and MAC433 (anti-GIMAP9), followed by horseradish-peroxidase conjugated goat anti-rat IgG, developed with Immobilon Western HRP substrate (MILLIPORE), and imaged using a G:Box (Syngene). (TIF) [file pone.0110294.s001.tif]

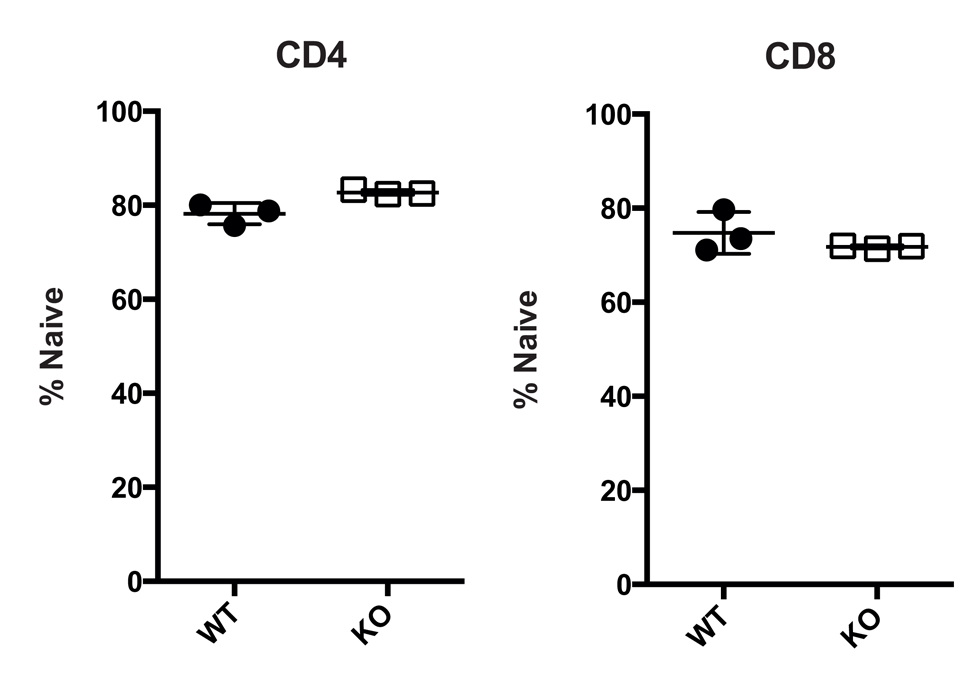

Supplement: Figure S2 — Proprtion of naïve T cells in periphery. Splenocytes from GIMAP8−/− mice and littermate controls were stained for CD4, CD8, CD44, and CD62L. Plots show proportion of naïve cells for individual mice (• indicates WT, n = 3, □ indicates GIMAP8−/−, n = 3). (TIF) [file pone.0110294.s002.tif]

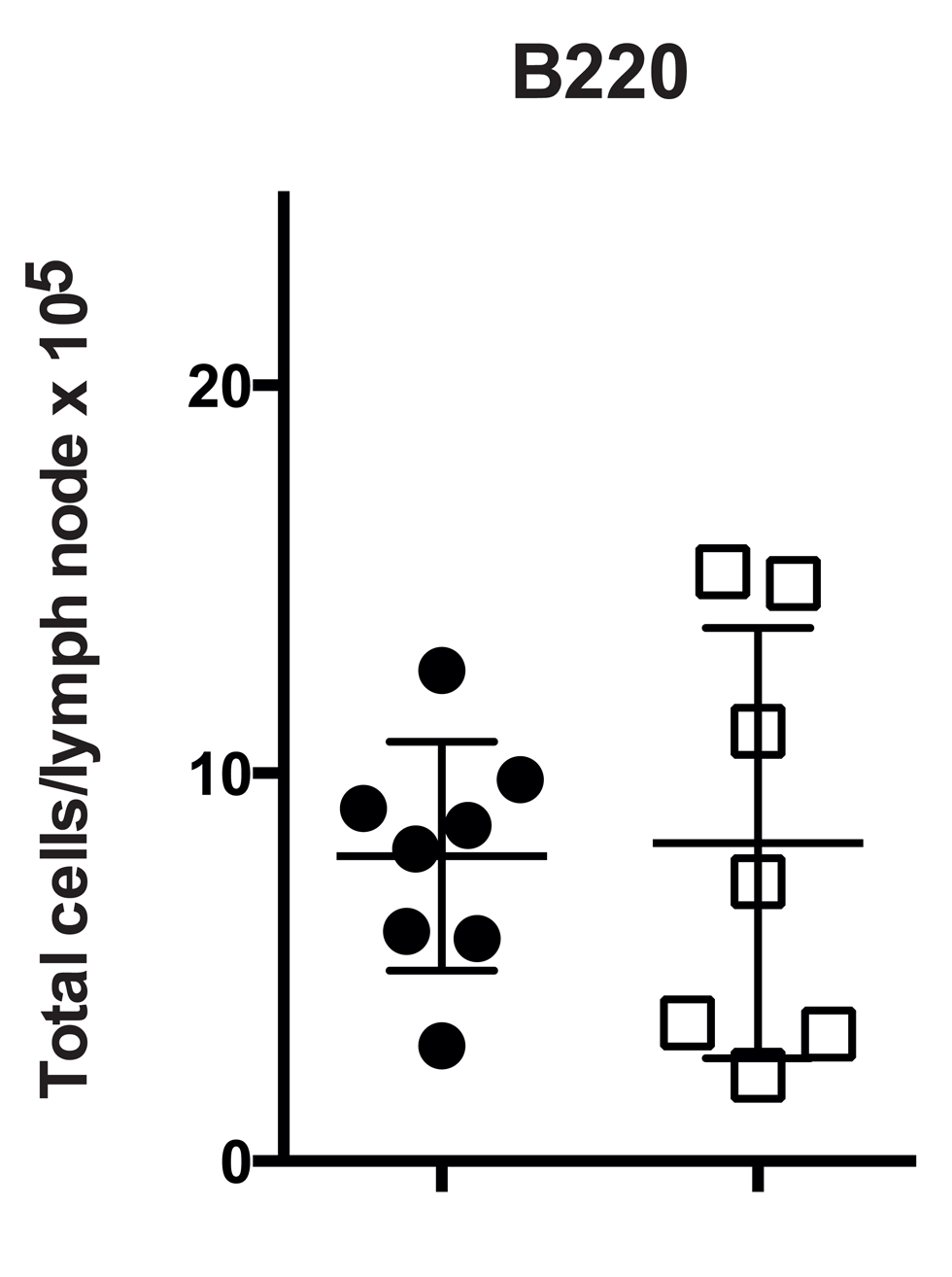

Supplement: Figure S3 — Numbers of B cells in lymph nodes. Cells from GIMAP8−/− mice and littermate controls were counted and stained for B220. Plots show number of cells for individual mice (• indicates WT, n = 8, □ indicates GIMAP8−/−, n = 7). (TIF) [file pone.0110294.s003.tif]

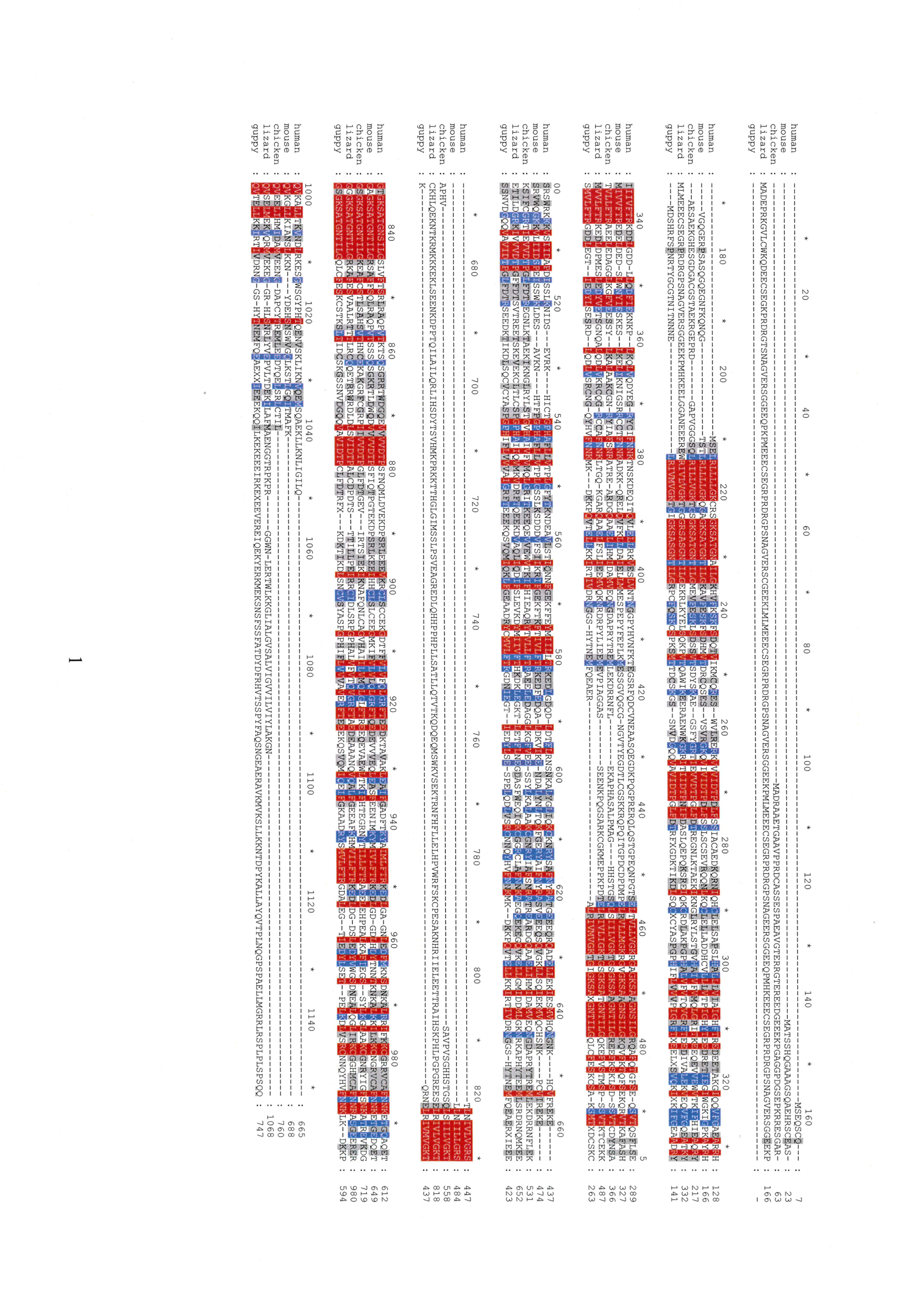

Supplement: Figure S4 — Alignment of GIMAP8 from various vertebrate classes. GIMAP8 protein sequences were aligned using ClustalW. The sequences had NCBI accession numbers: human (Homo sapiens) NP_783161.1; mouse (Mus musculus) NP_997651.1; lizard (Anolis carolinensis) XP_008106400.1; guppy (Poecilia reticulate) XP_008401341.1. The chicken (Gallus gallus) cDNA sequence was derived in-house by reverse transcriptase PCR from the DT40 cell line and then ab initio translated into protein. Sites at which all five proteins have identical or similar amino-acids are highlighted in red, four in blue and three in grey. G boxes are underlined in black. (TIF) [file pone.0110294.s004.tif]
